# Evaluations for In Vitro Correlates of Immunogenicity of Inactivated Influenza A H5, H7 and H9 Vaccines in Humans

**DOI:** 10.1371/journal.pone.0050830

**Published:** 2012-12-11

**Authors:** Robert B. Couch, William K. Decker, Budi Utama, Robert L. Atmar, Diane Niño, Jing Qi Feng, Matthew M. Halpert, Gillian M. Air

**Affiliations:** 1 Department of Molecular Virology and Microbiology, Baylor College of Medicine, Houston, Texas, United States of America; 2 Department of Pathology and Immunology, Baylor College of Medicine, Houston, Texas, United States of America; 3 Department of Medicine, Baylor College of Medicine, Houston, Texas, United States of America; 4 Department of Biochemistry and Molecular Biology, University of Oklahoma Health Sciences Center, Oklahoma City, Oklahoma, United States of America; Instituto Butantan, Brazil

## Abstract

**Background:**

Serum antibody responses in humans to inactivated influenza A (H5N1), (H9N2) and A (H7) vaccines have been varied but frequently low, particularly for subunit vaccines without adjuvant despite hemagglutinin (HA) concentrations expected to induce good responses.

**Design:**

To help understand the low responses to subunit vaccines, we evaluated influenza A (H5N1), (H9N2), (H7N7) vaccines and 2009 pandemic (H1N1) vaccines for antigen uptake, processing and presentation by dendritic cells to T cells, conformation of vaccine HA in antibody binding assays and gel analyses, HA titers with different red blood cells, and vaccine morphology in electron micrographs (EM).

**Results:**

Antigen uptake, processing and presentation of H5, H7, H9 and H1 vaccine preparations evaluated in humans appeared normal. No differences were detected in antibody interactions with vaccine and matched virus; although H7 trimer was not detected in western blots, no abnormalities in the conformation of the HA antigens were identified. The lowest HA titers for the vaccines were <1∶4 for the H7 vaccine and 1∶661 for an H9 vaccine; these vaccines induced the fewest antibody responses. A (H1N1) vaccines were the most immunogenic in humans; intact virus and virus pieces were prominent in EM. A good immunogenic A (H9N2) vaccine contained primarily particles of viral membrane with external HA and NA. A (H5N1) vaccines intermediate in immunogenicity were mostly indistinct structural units with stellates; the least immunogenic A (H7N7) vaccine contained mostly small 5 to 20 nm structures.

**Summary:**

Antigen uptake, processing and presentation to human T cells and conformation of the HA appeared normal for each inactivated influenza A vaccine. Low HA titer was associated with low immunogenicity and presence of particles or split virus pieces was associated with higher immunogenicity.

## Introduction

In a companion manuscript we reported a clinical trial of an inactivated subunit avian influenza A/H7N7 vaccine in healthy young adults that exhibited low immunogenicity despite vaccinations with two doses of up to 90 µg of the HA as determined in single radial immunodiffusion assays (SRID) [1]. This result prompted us to conduct some in vitro testing of this vaccine and some others in an effort to better understand the reasons for the low immunogenicity of unadjuvanted subunit avian influenza A virus vaccines in humans.

Avian influenza virus vaccines recently evaluated in humans have included types A (H5N1), A (H7N7) and A (H9N2). These evaluations have included subunit vaccines and whole virus vaccines with and without an adjuvant and a recombinant HA protein [2]–[30]. Noted early in the study of these vaccines without adjuvant was the tendency for them to induce lower antibody responses than was seen in humans with other subtype vaccines for novel viruses such as type A (H2N2) vaccines in 1957, type A (H3N2) vaccines in 1968, type A (H1N1) “swine” and “Russian” influenza vaccines in 1976 and 1977, and vaccines for the recently emerged influenza A (H1N1) virus from swine (2009 pandemic H1N1) [31]–[39]. Particularly notable were the relatively poor responses to the early A (H5N1) subunit vaccines, an early A (H9N2) vaccine (Atmar RL; personal communication) and in our trial with an A (H7N7) vaccine [2]–[12]. Each vaccine reportedly contained the specified dose of HA as detected in SRID tests, so low antigen dose was not incriminated as a cause for the low immunogenicity. It is notable that many of the early avian virus vaccines with low immunogenicity exhibited acceptable responses when given with an oil-in-water adjuvant [3], [8], [11], [14]. However, an adjuvant was not required for acceptable responses to the inactivated virus vaccines evaluated in 1957, 1968, 1976, 1977 and 2009 [31]–[39].

A summary of some of the antibody responses to subunit nonadjuvanted avian virus vaccines is shown in Table 1. The number of persons achieving a hemagglutination-inhibiting (HAI) titer of ≥1∶40 was the most consistently reported immune response permitting comparisons. Although a dose response was sometimes seen, two doses of some of the vaccines up to 90 µg per dose failed to induce the expected high response frequencies and levels of antibody in healthy adults. This contrasts to the high frequencies of responses to one dose of the pandemic A/California/09 (H1N1) virus vaccines in healthy adults and to the standard recommended two doses in young children (Table 1) [34]–[36]. Some of the avian virus vaccines were tested with and without an adjuvant. Alum as an adjuvant varied in induction of increases in responses; however, use of the adjuvants AS03 and MF59 uniformly resulted in major increases in response frequencies [3], [5], [7]–[9], [11], [13]–[23], [26], [27], [29].To try and understand the basis for the apparent immunizing deficiency of avian influenza virus vaccines without adjuvant, we sought alternative laboratory correlates for immune responses in humans. The findings of these efforts constitute the basis for this report.

**Table 1:** Percentage of Subjects Developing Serum Hemagglutination-inhibition Antibody Titers ≥1∶40 (or 1∶32) by Vaccine HA Dosage after Vaccinations with Monovalent Inactivated Influenza A Virus Vaccines.^1^

Subtype and Vaccine^2^	Mfr.^3^	% with Titer ≥1∶40 by Vaccine HA Dosage^4^						
		3.8 µg	7.5 µg	15 µg	30 µg	45 µg	90 µg	Ref.
H5N1								
Vietnam/04^5^	SP		6.5	17		34	46	2
Vietnam/04	GSK	17	16	35	43			3
Vietnam/04	SP			16		56		4
Vietnam/04	SP	2	0	13		26		5^8^
Vietnam/04	SP				35			6^8^
Vietnam/04	CSL		37	37				7
Vietnam/04	Novartis			24	18	29		8
Vietnam/04	SP (Lyon)		43	44	52			9
Vietnam/04^6^	SP						43	10^8^
Indo/05^6^	SP						51	10^8^
Indo/05	GSK	17						11
H7N7								
Mallard/00	SP		0	0		0	4	12^8^
H9N2								
HK/G1/99	GSK			67				13
HK/G1/99	Wyeth				11		15	-8
CK/G9/97	Novartis	67	58	50	75			14^8^
H1N1								
A/Cal/09 Vac	SP							
(ages 18–64 years)^7^			95	98	100			34^8^
(ages 3–9 years)			97	99				35^8^
(ages 0.5–3 years)			91	99				
A/Cal/09 Vac	CSL							36^8^
(ages 18–49 years)^7^				97	98			

1 All are after two doses of subunit vaccine 3–4 weeks apart and all trials were in healthy adults aged 18 to 49 except as noted.

2 Vaccine subtype and strain.

3 Manufacturer: SP – Sanofi Pasteur; GSK – GlaxoSmithKline, Wyeth – batch donated to NIAID.

4 Percents are for clinical trials reporting results for the dosages listed. Dosages are as determined in single radial immunodiffusion assays (SRID).

5 Data are FDA reanalysis of trial results reported in reference 2.

6 Data provided by Belshe, RB, et al. (ref 10).

7 Data are after one dose for ages 18–64 and 18–49 and after two doses for 3–9 and 0.5–3.

8 Clinical trial results for vaccines used in this study, references 5, 6, 10 were with the USA licensed H5/VN/04 vaccine.

## Materials and Methods

### Vaccines and hemagglutinin (HA) proteins

Vaccines used in these studies were all obtained from the National Institute of Allergy and Infectious Diseases, U.S.A. Monovalent inactivated subunit avian influenza A vaccine lots used for clinical trials in humans (Table 1) and in the in vitro studies reported here were: A/Vietnam/1203/04 (H5N1) (A/VN/04/H5), A/Indonesia/05/05 (H5N1) (A/Indo/05/H5), A/Mallard/Netherlands/12/2000 (H7N7) (A/Mallard/00/H7), A/Hong Kong/1073/G1/99 (H9N2) (A/HK/G1/99/H9), and A/Chick/Hong Kong/G9/97 (H9N2) (A/Chick/G9/97/H9). Two recent influenza A subunit vaccine lots also used for clinical trials in humans and evaluated in the in vitro studies reported here were both monovalent inactivated A/California/07/09 (H1N1) (A/Cal/07/09/H1) vaccines for the 2009 pandemic with influenza A (H1N1).

Hemagglutinin (HA) proteins were obtained from BEI Resources. They were recombinant proteins produced in SF9 insect cells using a baculovirus expression system. HA proteins used were from the same viruses used to prepare the vaccines used in the clinical trials and by us in our in vitro studies; they were from A/VN/04/H5, A/Indo/05/H5, A/HK/G1/99/H9 and A/Chick/G9/97/H9. The H7 and H1 HA, however, were from A/Netherlands/219/03 (H7N7) (A/Neth/219/03/H7) and A/California/04/09 (H1N1) (A/Cal/04/09/H1).

### Viruses

Viruses used for comparison to vaccines in conformation assays were the vaccine virus seeds of A/VN/04/H5 and A/Mallard/12/00/H7. These vaccine virus seeds and the A/Cal/04/09/H1 virus were provided by the Centers for Disease Control (CDC).

Viruses used for H9 comparisons were A/Quail/Hong Kong/G1/97 and A/Chick/G9/97/H9, each reassorted with A/Seal/Mass/1/80; each reassortant virus is H9N7 (provided by R. Webster, St. Jude Children's Research Hospital, Memphis, TN, USA).

### Antisera

Polyclonal antisera obtained from BEI Resources were A/VN/04/H5 goat antiserum prepared against bromelain-released HA (NR-2705), goat antisera prepared against baculovirus-expressed HA from A/Neth/219/03/H7 (NR-9226), A/Chick/G9/97/H9 (NR-668) and A/Cal/04/09/H1 (NR-15696) and sheep antiserum prepared against baculovirus-expressed HA of A/HK/G1/97/H9 (NR-662). Monoclonal antibodies from BEI Resources were mouse ascites fluid containing anti-HA antibody against A/VN/04/H5 (NR-2743) and A/Duck/Hong Kong/Y280/97 (H9N2) (NR-9491). Anti-HA monoclonal antibody 29E3 which was prepared against A/Cal/04/09/H1, was provided by A Garcia-Sastre, Mt. Sinai School of Medicine, New York, NY, USA.

### Antigen processing and presentation

**Figure 1:**
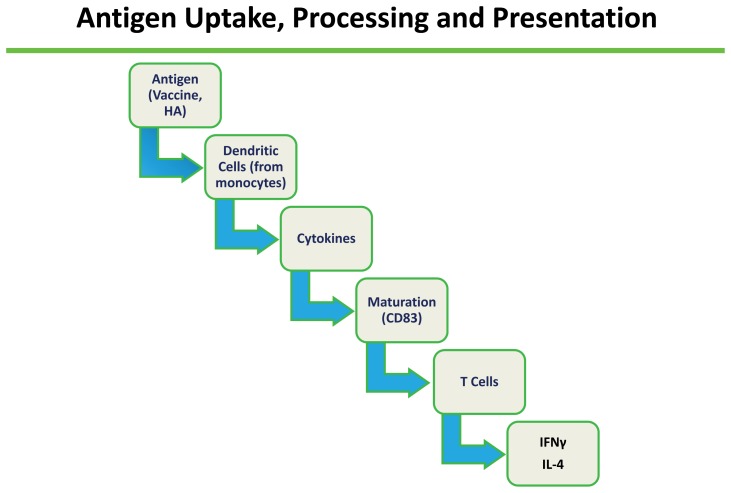
Evaluation sequence of antigen uptake, processing and presentation of influenza A vaccine and HA antigens to human T cells.

Selected vaccine and HA proteins were tested for antigen processing and presentation by dendritic cells (DC) to T cells for eliciting immune responses (Figure 1). Immature dendritic cells were generated from discarded normal donor apheresis or white blood cell buffy coats for six days with 50 µg/ml of GM-CSF and 10 µg of IL-4 and then loaded for three hours with human influenza vaccine containing 10–15 µg of the HA protein or 10–15 µg/ml of recombinant HA protein. Antigen loaded DC were matured for two days using a cocktail of inflammatory cytokines [40]. The cytokine cocktail consisted of 50 ng/ml of GM-CSF, 10 ng/ml of IL-4, 10 ng/ml of IL-1beta, 15 ng/ml of IL-6, 10 ng/ml of TNF alpha and 1 µg/ml of PGE_2_. DC were analyzed for maturation status by flow cytometry and then used to prime autologous T-cells. Immature dendritic cells were characterized by absent expression of CD80, absent expression of CD83, highly variable expression of CD86, and HLA-DR. Mature dendritic cells were characterized by expression of both CD80 and CD83 as well as more uniform, high-level expression of CD86 and HLA-DR. For antigen presentation to T cells, 10^6^ DC were incubated with 10^7^ autologous lymphocytes (1∶10) for nine days. Excess DC was cryopreserved to be used for repeat stimulation. Exogenous IL-2 was given at a concentration of 200 U/ml on days 5 and 7. On day nine, lymphocytes were restimulated with cryopreserved DC at a ratio of 1 DC per 10 lymphocytes. Lymphocytes were given exogenous IL-2 on day 10, and interferon gamma (IFNγ) and IL-4 ELISpot analysis was performed on day 12. For the ELISpot assay, lymphocytes were plated in triplicate wells at a concentration of 50,000 cells per well. Assays used ELISpot plates (BD Biosciences) and were performed as described [41].

### ELISA for native and denatured antigen

The reactivities of antibodies with vaccines and purified virus preparations were compared under native and denaturing conditions. The virus or vaccine was captured on wells coated with fetuin (50 µl of 400 µg/ml in PBS overnight at 4°C); functional, correctly folded HA binds to the fetuin sialic acid but denatured HA has no sialic acid binding site and does not bind to fetuin [42]. The captured native HA was measured using serial dilutions of polyclonal antiserum or a monoclonal antibody (mAb) to obtain the “native HA” binding curves. Duplicate wells were treated with 80 µl of methanol prewarmed to 60°C then incubated at 37°C for 30 minutes to unfold the bound HA before adding polyclonal antiserum or mAb. This allows for measurement of antibodies against denatured HA in the sera. Most neutralizing monoclonal antibodies are specific for native protein but some will cross-react with unfolded protein. Neutralizing antisera always contains a mixture of antibodies specific for native or unfolded protein. This native vs. unfoldon ELISA is used to separately measure those classes of antibodies and so obtain a comparative measure of quality of the original antigen. The native and unfoldon binding curves were processed using Prism software to obtain the relative amount of binding (Bmax) and the avidity (Kd). To compare results across the different subtypes of HA and different states of HA (whole virus or vaccine), the results for both Bmax and Kd were normalized to the values for native protein.

### SDS PAGE and western blot

The vaccine (0.9 µg protein) and virus samples (diluted to approximately the same concentration of the influenza virus M1 protein) were mixed with standard gel loading buffer (0.2 M tris, pH 6.8, 20% glycerol, 10% SDS, 0.05% bromophenol blue, 10 mM β-mercaptoethanol, boiled 3 min) or non-reducing gel loading buffer (in which β-mercaptoethanol and the boiling step were omitted), and run on 10% polyacrylamide gels without a stacking gel. The gels were either stained with Coomassie blue or blotted onto a PVDF membrane and probed with a mixture of antisera representing each subtype HA on the gel. The blot was developed with a mixture of alkaline phosphatase-conjugated anti-goat, rabbit and sheep secondary antibodies and developed with NBT/BCIP substrate [Pierce Biotechnology, Inc.].

### Hemagglutination tests

Each vaccine was tested for hemagglutinating activity using serial two-fold dilutions in 96-well plates with PBS as diluent. Red blood cells (rbc) used were turkey (0.5%), chicken (0.5%) and horse (1% in 0.5% BSA). Comparisons were simultaneous and titers were the last dilution providing complete hemagglutination.

### Electron microscopy

Vaccine dilutions were absorbed onto homemade parlodion/carbon-coated glow-discharged 400 mesh EM grids. The negative staining solution used was 1.5% *Phosphotungstic acid* (PTA) at pH 7.0. Images of stained virus particles in vaccines were captured with a JEOL JEM 1230 80 kV transmission electron microscope. All negative stained samples were observed with 5K up to 40K magnifications and representative images were taken from the grids for each sample using Gatan Image software.

## Results

### Evaluations

For evaluations, we included vaccine lots that were used for immunogenicity trials identified in Table 1, their HA proteins, their seed viruses, and p2009 (H1N1) vaccines and HA. The A/Vietnam/04 (H5N1) vaccine used for the in vitro evaluations was from the licensed vaccine used for the clinical trials reported in references 5, 6 and 10. The A/Indo/05 (H5N1), A/Mallard/00 (H7N7) and one of the A/Calif/09 (H1N1) vaccines were the vaccines used for the trials reported in references 10, 12, 34 and 35; each vaccine was prepared by Sanofi Pasteur (SP). The A/HK/G1/99 (H9N2) prepared by Wyeth Laboratories and the A/CK/G9/97 (H9N2) vaccine prepared by Novartis that were used for our in vitro studies were also used for the clinical trials reported in Table 1 for the Wyeth prepared vaccine (no reference) and the Novartis vaccine (reference 14). A second A/Calif/09 (H1N1) vaccine, also used for in vitro evaluations, was a vaccine used in the clinical trial reported in reference 36. The viruses and HA proteins were those listed in Materials and Methods.

Evaluations performed to assess for normality were (1) vaccine and HA antigen uptake, processing and presentation by human dendritic cells to T cells, and (2) assessing the conformation of the HA antigens in antibody binding assays and gel analyses. Comparative evaluations of vaccine preparations were (1) HA titers with different animal red blood cells, and (2) vaccine morphology in electron micrographs.

### Antigen processing and presentation

**Figure 2:**
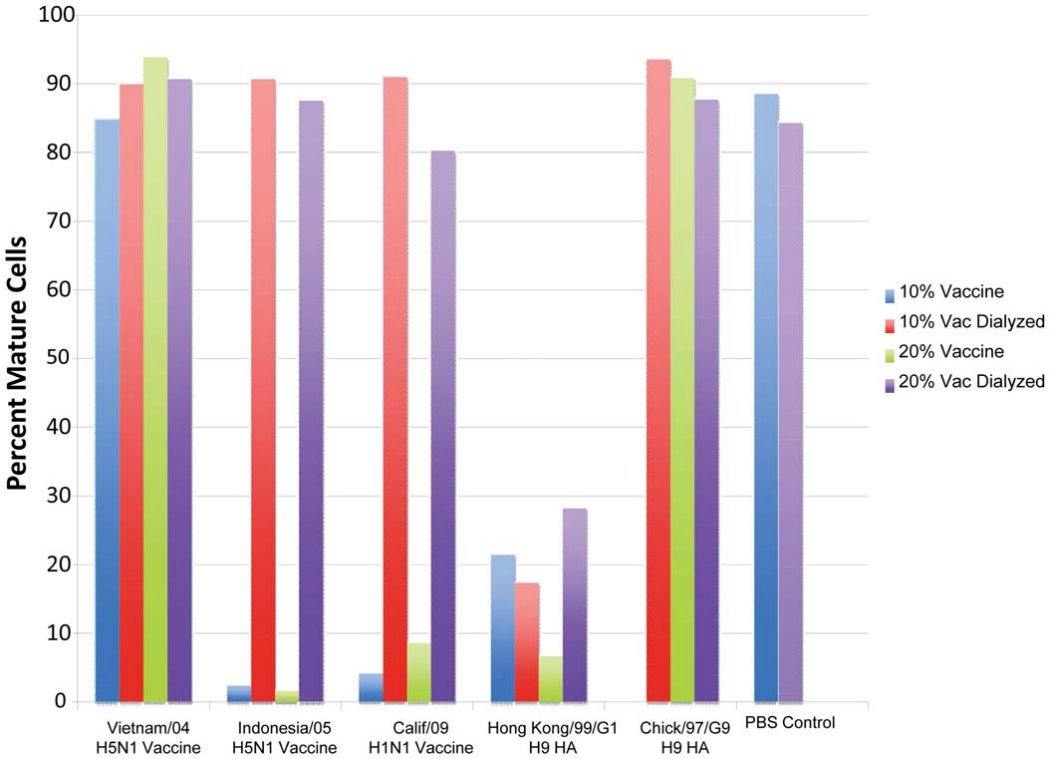
Percent of human dendritic cells maturing after influenza A antigen loading. Data are before and after dialysis with 10 and 20% of the initial vaccine or HA amount used so as to reduce any inhibitory effect. The vaccine concentration should not affect maturation; maturation in the presence of vaccine should be similar to the PBS control. The PBS control is also before and after dialysis. Available vaccine was insufficient for testing all four variables for the HA of Hong Kong/G1/99 (H9N2).

After generating immature dendritic cells from peripheral blood of healthy adults, they were loaded with an influenza A vaccine or rHA. Dendritic cells were then matured with the described cocktail of cytokines (see M&M) and analyzed for maturation by flow cytometry (CD83). Initial maturation testing indicated full maturation in three of the five vaccine tests and in five of the six HA preparation tests (Table 2). Dialysis was performed on the antigen preparations where DC did not mature with a repeat of testing before and after dialysis. The A/Vietnam/04 (H5N1) vaccine used in other in vitro comparative testing was included in the repeat testing. Maturation of DC with vaccine or HA in the repeat test is shown in Figure 2. The initial maturation failure of the H5N1 A/Indo/05 and H1N1 A/Calif/09 vaccines was verified as shown; the inhibition was removed by dialysis of vaccine before retesting. The dialyzed inhibitory component was probably thimerosal preservative. Maturation with A/VN/04 (H5N1) vaccine (not tested initially) occurred before and after dialysis as shown. The after dialysis results in the repeat testing are summarized in Table 2. The A/HK/G1/99 (H9N2) recombinant HA had inhibited maturation initially; maturation occurred in the repeat testing although responses were low and not improved with dialysis. The reason for the poor maturation with this preparation is unknown; an alternative lot for testing was not available. Maturation of DC with the recombinant HA of A/CK/G9/97 (H9N2) appeared normal as in the initial tests (Table 2). Thus, except for the low responses for the A/HK/GI/99 rHA, dendritic cell maturation in the presence of vaccine or HA appeared normal (Table 2, Figure 2).

**Table 2:** Dendritic Cell Maturation and Cytokine Secretion by T Cells Stimulated in vitro with Influenza A Virus Vaccines and Hemagglutinin Proteins.^1^

	Maturation^3^		IFNγ^4^		IL-4^4^
Stimulator^2^	Initial Test	After Dialysis^5^	Initial Test	After Dialysis^5^	Initial Test
**Vaccine**					
H5N1					
A/VN/04 (SP)	NT^6^	Yes	NT^6^	>10^5^	NT^6^
A/Indo/05 (SP)	No	Yes	0	>10^5^	0
H7N7					
A/Mallard/00 (SP)	Yes		>10^5^		10^4^
H9N2					
A/HK/G1/99 (Wyeth)	Yes		>10^5^		>10^4^
A/CK/G9/97 (Novartis)	Yes		>10^5^		>10^4^
H1N1					
A/Cal/09 (CSL)	No	Yes	0	>10^5^	0
**rHA**					
H5N1					
A/VN/04	Yes		>10^4^		>10^4^
A/Indo/05	Yes		>10^4^		>10^4^
H7N7					
H7N7 A/Mallard/00	Yes		>10^4^		>10^4^
H9N2					
A/HK/G1	No	Yes (low)	0	>10^4^	0
A/CK/G9	Yes	Yes	>10^4^	>10^4^	>10^4^
H1N1					
A/Cal/09	Yes		>10^5^		>10^4^

1 Immature dendritic cells loaded with antigen, matured with cytokines and used to present antigens to T cells that secrete cytokines.

2 Monovalent vaccine (manufacturer) or recombinant HA proteins described in M&M.

3 As determined in FACS for CD83 and HLADR.

4 Numbers of ELISpots for indicated cytokine secretion after T cell stimulation; unstimulated lymphocytes, DC only and medium controls did not show ELISpots.

5 The test was before and after dialysis in a repeat test, after dialysis result shown.

6 NT = not tested.

Presentation of antigen by mature DC to T cells was evaluated by T cell cytokine secretion; results are summarized in Table 2. Each of the DC preparations with vaccine and HA that matured also induced T cells to secrete interferon gamma (IFNγ) and IL-4 as shown in cytokine Elispots whereas those DC with vaccines and the HA preparation that did not mature in initial testing failed to induce T cell secretion of IFNγ and IL-4 (Table 2). However, the two vaccines [A/Indo/05 (H5N1) and A/California/09 (H1N1)] that inhibited maturation before dialysis but not after dialysis, induced IFNγ in the repeat test after dialysis. Although maturation was impaired in the initial and post dialysis tests with the rHA preparation of A/HK/G1/99 (H9N2), IFNγ secretion in the after dialysis test was similar to the other HA preparations. DC maturation and cytokine secretion with the A/VN/04 (H5N1) vaccine was similar to the other vaccines. Thus, these studies indicated that antigen processing by DC and presentation of avian influenza virus vaccine antigens and their hemagglutinin proteins by human DC to T cells appeared normal.

### Conformation of the HA antigens

**Figure 3:**
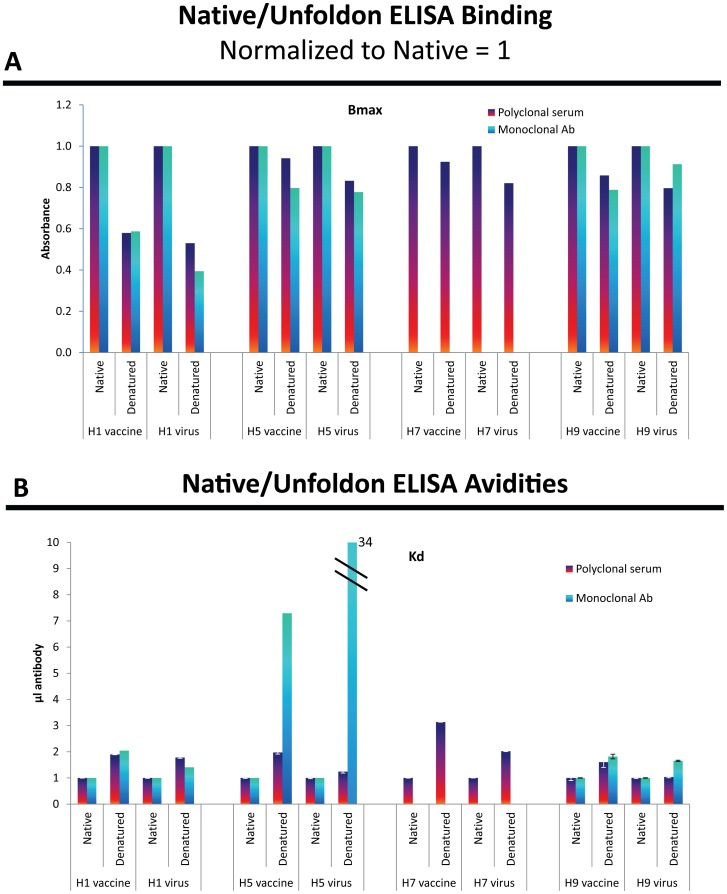
Native and unfoldon ELISA assays for viruses and vaccines using polyclonal antisera and monoclonal antibodies. The viruses and vaccines were diluted to equivalent hemagglutination units (HAU) although not comparable for detergent-released HA in vaccines versus virus particles and the H7 vaccine had no HAU. Analysis of the binding curves gives total binding sites (Bmax) (Figure 3A) and overall avidity (Kd) (Figure 3B).

ELISA Analyses. The ELISA analyses showed decreased reactivity (Bmax) after denaturation for both viruses and vaccines (Figure 3A and 3B). Monoclonal antibodies that are conformation-specific often show no binding after denaturation [42]. The mAbs used in the present study show considerable binding to denatured protein with the H1 mAb showing the most discrimination at about half the Bmax of native binding and the H5 and H9 mAbs showing only about a 20% decrease in Bmax after denaturation of the virus or vaccine. The affinities were dramatically lower (higher Kd) after denaturation for the H5 mAb while the H1 and the H9 mAbs showed only 2-fold or less change in affinity. While the discrimination between native and unfolded protein is less with these antibodies than others we have used [42], [43], the proportions of the anti-native and anti-unfoldon reactivities are similar for both vaccine and virus.

Because of lacking a mAb for H7 that gave an HAI titer, the H7 evaluation could only be done with polyclonal serum; these results are also shown in Figure 3. A polyclonal antiserum that inhibits hemagglutination will contain antibodies that bind to native protein and to unfoldons. We cannot predict the proportions, as the unfoldon ELISA might give a signal that is lower, higher, or equal to the native ELISA signal. All the antisera tested gave higher Bmax with native antigen than with the denatured antigens, indicating that the population of anti-unfoldon antibodies is small. The affinities were higher (lower Kd) for native than for unfolded protein for all antisera except for the H9 antiserum with H9 virus. The differences between Bmax for native HA and unfoldons varied from two-fold for H1 to 10–20-fold for the other sera; the Kd showed greater differences, especially for H7. The polyclonal sera and mAbs gave similar ratios of native and unfoldon reactivities for purified virus and vaccines for both Bmax and Kd (Figure 3).

In the absence of mAbs that show good discrimination between native and unfolded antigen it is not possible to assess the ratio of unfolded to native HA in each preparation. Even the H7 vaccine that did not hemagglutinate red cells (see later) is not very different in native/unfoldon reactivity to the purified virus but confirming that they are similar will require a conformation-specific mAb.

**Figure 4:**
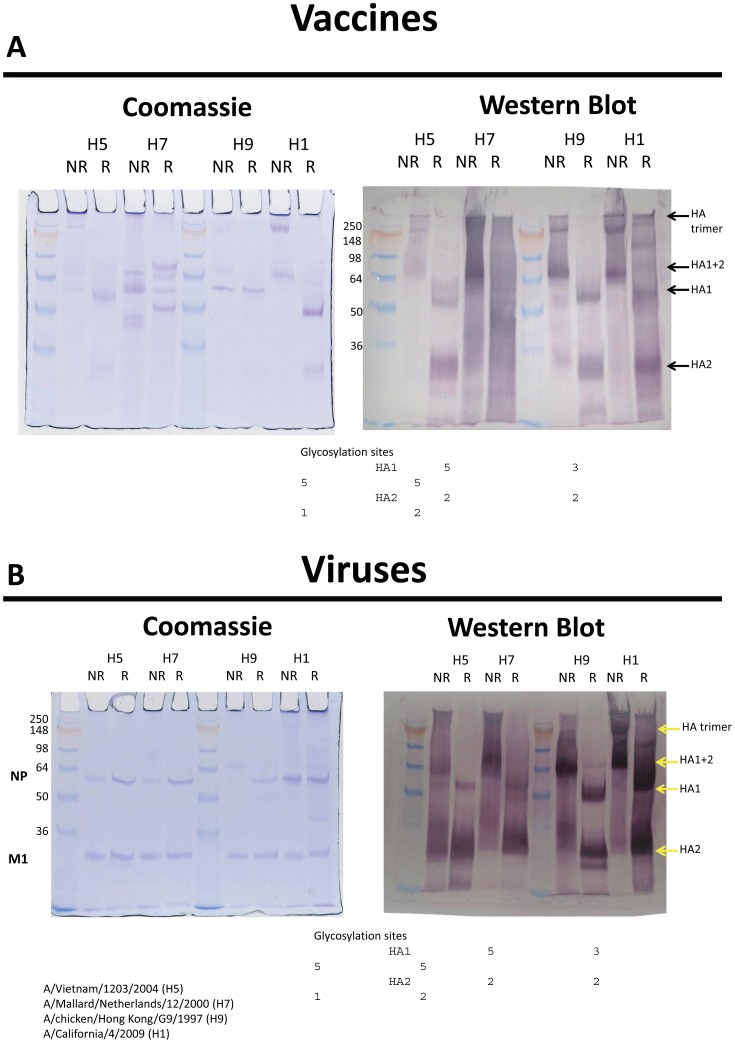
Polyacrylamide gels run under reducing and nonreducing conditions for trimeric HA and HA1 and HA2 subunits bound and separate. Coomassie blue stain was used for protein and western blots with polyclonal antisera for protein identity. Gels are for vaccines (Figure 4A) and viruses (Figure 4B). Baculovirus expressed H3 HA was used as control (not shown).

Gel Electrophoresis and Western Blot Analysis (Figures 4A and 4B). Although the HA trimer is not stabilized by disulfide bonds, a band corresponding to trimeric HA can be seen on an SDS gel when the sample has not been reduced or boiled. Glycoproteins stain poorly and the bands are diffuse, so they are better visualized by Western blot. Figure 4A shows an example of a stained gel of the H1, H5, H7 and H9 vaccines and the corresponding blot probed with anti-HA antiserum. In the non-reduced lane we expect to see trimer plus a band corresponding to the disulfide-linked HA1+HA2 (i.e. equivalent to uncleaved HA). Although the amount of trimer seen on the SDS gel is variable and not quantitative, it is clear for the H1 vaccine on the Coomassie-stained gel, along with the HA1+2 band. When reduced, only HA1 and HA2 bands are seen. The Western blot of the H1 vaccine shows the same pattern but with additional higher molecular weight bands of unknown origin. In the H9 vaccine, the stained gel shows only NP clearly, but the Western blot confirms the presence of trimeric HA and HA1+2 in the non-reduced lane and HA1 and HA2 bands in the reduced lane. The H5 vaccine appears similar, but with an additional band on the Western blot that probably reflects heterogeneity in glycosylation of HA1.

The H7 vaccine shows multiple bands on Coomassie staining and, while there are candidate bands for HA1+2 and HA1 in the non-reduced and reduced lanes respectively, there is no HA2 band and overall the pattern is very different compared to the other vaccines, particularly the relationship between non-reduced and reduced bands. The Western blot of the H7 vaccine shows considerable smearing, and repeated attempts to clarify the pattern using different conditions and dilutions of antiserum failed to resolve clear bands. The smearing is not entirely a property of the antiserum because the whole blot was treated with a mixture of all the antisera, and the other lanes are relatively clear.

When purified virus is run under similar conditions (Figure 4B), the stained gel shows NP and M bands with only traces of HA (visible in the H9 lanes). The Western blot shows the HA1+2 band for each virus in the non-reduced lane, and HA1 and HA2 bands in the reduced lanes. In the whole virus, HA trimer is not clearly seen, probably due to incomplete dissociation of the virion under the non-boiling conditions. Although not a definitive evaluation, no abnormalities were noted in the conformation of the HA in the vaccines including the H7 vaccine.

### Hemagglutination titers

Shown in Table 3 for the different vaccines evaluated are the HA concentrations in SRID assays and corresponding titers in hemagglutination tests with three different red blood cell sources. For comparability, concentration of each vaccine was adjusted to make each as similar as possible by SRID. Notable in the table is the considerable variation in titers with the different red cell sources. No HA activity was detected for the A/Mallard (H7N7) vaccine with any of the different sources of red cells. Highest titers were obtained with turkey red cells and lowest with horse red cells. Horse red cells are reported to be superior to turkey red cells for antibody measurements for A/H5N1 and some other avian viruses but only two horse cell lots were tested and HAI assays for antibody with horse cells were not performed [44], [45]. The same pattern of titers among vaccines was seen with chicken red cells as with turkey red cells but titers were lower. Despite lower HA titers than some of the other vaccines, the A/H1N1 vaccines had appeared to be more immunogenic in humans (Table 1). The lowest immunogenicity was seen among subjects given the A/Mallard (H7N7) and the A/HK/G1/99 (H9N2) vaccine and those two vaccines had the lowest HA titers. A test for a significant correlation between the HA titer and immune response frequencies was not significant (Spearman rank test, r = .343, p>.10) but the number of test entries was small.

**Table 3:** Hemagglutination Titers of Monovalent Inactivated Influenza A Avian and Pandemic 2009 Influenza A Vaccines.

		Hemagglutination Titer with Indicated Red Blood Cells		
Influenza A Subtype & Vaccine	HA µg/ml^1^	Turkey^2^	Chicken	Horse^3^
H5N1				
Vietnam/04 (SP)	30	10,720	1024	128
Indo/05 (SP)	60	21,400	4096	192
H7N7				
Mallard/00 (SP)	60	<4	<4	<4
H9N2				
HK/G1/99 (Wyeth)	60	661	64	<4
CK/G9/97 (Novartis)	60	35,500	≥16,384	4
H1N1				
Cal/09 Vac (SP)	30	4370		
Cal/09 Vac (CSL)	60	7080	2048	<4

1 Concentrations in single radial immunodiffusion (SRID) assays as reported to NIAID.

2 Mean of three tests.

3 Mean of two tests performed as recommended by Stevenson, et al. [43].

### Electron micrographs

**Figure 5:**
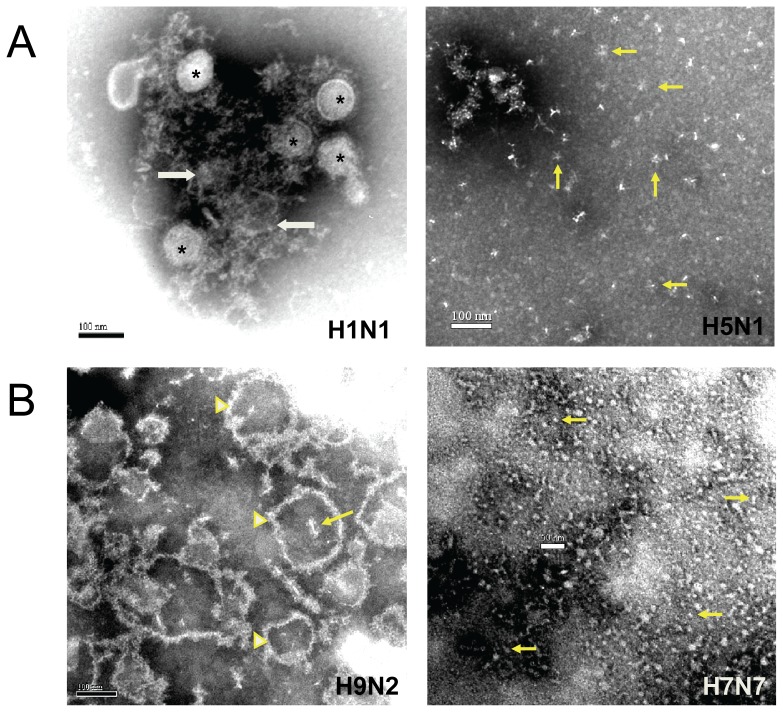
Selected electron micrographs of vaccines illustrating the morphologic structures described in **Table 4**. Figure 5A shows intact and split virus particles (asterisks) in the influenza A/Cal/04 (H1N1) subunit vaccine (CSL) of Table 1 along with structures of indistinct morphology (arrows). Also shown in Figure 5A is an EM of the A/Vietnam/04 (H5N1) subunit vaccine (SP) of references 5, 6, 10 in Table 1 that was selected to show a large number of stellate structures (arrows point to examples) although indistinct structures similar to those in the H1N1 vaccine were dominant in the H5N1 vaccine (not shown). Figure 5B is an EM of the influenza A/CK/G9 (H9N2) vaccine (Novartis) of Table 1 that shows the predominant varying size particles of membrane with external projections as well as a number of stellates, one apparently within an empty particle (arrow). Also in Figure 5B is an EM of the influenza A/Mallard (H7N7) vaccine (SP, ref. 12 in Table 1) that primarily showed small (5 to 20 nm) round and elongated structures (arrows point to examples). Some stellates and a rare intact particle were also seen.

Electron micrographs were obtained for comparing morphology of the influenza vaccine virus in the seven clinical trial vaccines evaluated for HA titer (Table 3) and included those evaluated for antigen processing and for antibody binding. All EMs at varying magnifications (X5000 to 40,000) for all vaccines were first reviewed for the different types of structures seen. Intact virus particles, large and small pieces of split virus, stellate structures, tiny (5–20 nm) round and elongated structures and structures with indistinct morphology were the different morphologic structures seen (Table 4). Representative micrographs are shown in Figures 5A and 5B. The electron microscopist then reviewed the EMs for each vaccine and described the occurrence of each structure on a 0 to 4 scale of abundance for each vaccine. For the review, the electron microscopist knew the vaccine virus but had no knowledge of the immunogenicity of the preparation in humans. Results for each vaccine are shown in Table 4. The two pH1N1 2009 vaccines were the most immunogenic and the A/CK/97/G9/97 (H9N2) vaccine immunogenicity was moderately-high. Each of these vaccines contained a large number of virus particles, split virus pieces of varying size or discrete particles of varying sizes with circumscribed membranes and external projections but apparently empty contents [see EM for A/CK/G9/97 (H9N2)]. Much smaller units including stellate structures and tiny (5–20 nm) round and elongated structures were notable in the vaccines of lower immunogenicity. The least immunogenic vaccines [A/Mallard (H7N7) and A/HK/G1/99 (H9N2)] primarily exhibited the small 5–20 nm structures. There was a statistically significant difference between the percent of subjects with titers ≥1∶40 who had received a vaccine with a score of ≥3 for particles or split virus pieces and the percent for those who received the other vaccines (Mann-Whitney U, p = .02). The relative abundance of structures of indistinct morphology did not appear to relate to immunogenicity.

**Table 4:** Relative Proportions of Different Morphologic Units Seen in Electron Micrographs of Monovalent Influenza Vaccines.

	Morphology^1^				
Subtype & Vaccine	Intact Particles	Split Virus	Stellates	5–20 nm Structures	Indistinct Structures
H5N1					
Vietnam/04 (SP)	0	1	3	1	4
Indo/05 (SP)	0	1	1	1	4
H7N7					
Mallard/00 (SP)	<1	0	1	4	2
H9N2					
HK/G1/99 (Wyeth)	1	0	1	3	1
CK/G9/97 (Novartis)	4	1	2	1	0
H1N1					
Cal/09 Vac (SP)	0	4	2	2	2
Cal/09 Vac (CSL)	3	2	1	1	3

1 Scale of 0 to 4; 0 = none seen, 4 = abundant; <1 = rare.

## Discussion

The present studies were prompted by the very poor immunogenicity in humans of an inactivated monovalent subunit influenza A (H7N7) vaccine despite SRID values indicating the HA dosages given were those that should induce good serum antibody responses [12]. Similar antigen dosages have regularly induced good antibody responses with seasonal vaccines [46], [47]. The A/H7N7 vaccine was developed as a potential vaccine for one of the avian influenza A viruses thought to be a possible cause of an influenza pandemic. Monovalent inactivated avian influenza A virus vaccines have been prepared by various manufacturers for influenza A subtypes considered likely causes of a pandemic and tested for safety and immunogenicity in humans. These studies have focused on influenza A (H5), (H7) and (H9) [2]–[30]. The NA in these vaccines has usually been N1 or N2 but the NA in the H7 vaccine we tested was N7. In general, those avian virus vaccines without adjuvant have exhibited poor immunogenicity in humans (see Table 1) despite the fact that all were prepared by manufacturers using their standard methods for preparing seasonal influenza vaccines. All were prepared from virus grown in embryonated chicken eggs but a study not in the table with an influenza A (H7N1) vaccine made in a tissue culture system also exhibited low immunogenicity [26]. As indicated earlier, this low immunogenicity was not the experience in 1957, 1968, 1976, 1977, and 2009 with monovalent inactivated influenza vaccines for new subtypes or major new variants of influenza A viruses that emerged and spread worldwide [31]–[36].

One solution for correcting the low immunogenicity of these vaccines has been to give them with an oil-in-water adjuvant. Alum as an adjuvant for the avian virus vaccines has not reliably improved antibody responses but oil-in-water adjuvants have, so far, shown consistent improvement in responses [3], [5], [7]–[9], [11], [13]–[23], [26], [27], [29]. While the adjuvant remedy for the low immunogenicity of avian influenza A vaccines prepared using seasonal vaccine methods is available, understanding the reason for the low immunogenicity of these nonadjuvanted vaccines is desirable. For this reason, we undertook a series of studies of the vaccines to seek some understanding.

The first steps leading to an immune response are uptake, processing and presentation of antigenic determinants to T cells. We evaluated this sequence using established in vitro methods. We used available monovalent vaccines of H5, H7, and H9 that had been used in clinical trials and we also included a recent 2009 pandemic H1N1 vaccine which was highly antigenic in clinical trials in primed adults as a single dose and in unprimed children in standard two dose schedules [34]–[36]. Since the content of vaccines can include proteins other than the HA, we included purified recombinant HA proteins expressed in a baculovirus system in the studies. In these studies, avian influenza vaccine and HA antigen uptake, processing and presentation to human T cells for initiating an immune response appeared normal.

In view of the immunogenicity reported and the enhancement with some adjuvants, it seemed unlikely that there was a general defect in the conformation of the avian HA in the vaccines although variation and a defect in the HA in the H7 vaccine seemed possible. To evaluate the conformation of the HA antigens, we tested the vaccine HA protein interactions with antisera in ELISA assays and in gel electrophoresis and western blots. Interactions with HA proteins in ELISA assays were evaluated for polyclonal and monoclonal antisera against native and denatured (unfoldon) proteins for each vaccine and for a matching virus. No differences in the ratio of native/unfoldons between the virus and the vaccine for each vaccine and virus HA, including the H7 vaccine, were detected; however, reagents for determining the actual ratios were not available.

HA trimers were detected in the H5, H9 and H1 but not in the H7 western blots; however, trimers in the H7 vaccine could not be excluded as the antiserum showed considerable background and the Coomassie stained gel pattern was not interpretable. Although not a definitive evaluation, no abnormalities in the state of the HA in the various inactivated avian influenza virus vaccines, including the H7 vaccine, were identified.

In order for vaccine virus to hemagglutinate red blood cells (RBC), the HA must exhibit the biological activity of binding to the receptor on RBCs and be in a morphologic configuration that can bridge to other RBCs to induce hemagglutination. The requirements for this are intact conformational HA that can bind to receptor and a morphologic structure with a number of HA units such as a virus particle that can bridge between RBCs and lead to hemagglutination. Before SRID was adopted as the standard for HA quantitation in vaccines, vaccine antigen quantitation was done using hemagglutination and was expressed as HA units or chick cell agglutinating units (CCA) [1], [48]. In hemagglutination titer comparisons (Table 3), turkey RBCs were most sensitive, chicken RBCs exhibited the same pattern as turkey cells but with lower titers and horse red cells were lowest. Horse RBCs are reported to exhibit higher titers of anti-HA antibody for the avian viruses than either avian RBC despite requiring more virus per HA unit [43], [44]. Notable in these comparisons was the poor correlation between the HA quantity in SRID assays and the HA titer with RBCs and the complete absence of hemagglutination for the H7 vaccine despite a SRID concentration of 60 µg/ml. The H7 vaccine with no HA titer was the poorest immunogen in humans and the HK/G1/99 (H9N2) vaccine with very low titers was next poorest. The highest HA titer was exhibited by the CK/G9/97 (H9N2) vaccine and it appeared to be the best immunogen among the avian vaccines.

The evaluations of morphology of the various subunit vaccines suggested an association with immunogenicity of the vaccines in humans. The best immunogenicity in humans was exhibited by the vaccines that contained residual virion particles, particle-like structures, or pieces of viral particles of varying sizes, some of which are large pieces clearly containing surface structures corresponding to the HA and NA; these vaccines were the two p2009 (H1N1) vaccines and the CK/G9/97 (H9N2) vaccine. This finding is similar to that of the many inactivated trivalent seasonal vaccines we have viewed and reported by others [49]. The H7 vaccine morphology was primarily small structural units in the size range of the HA and NA glycoproteins. Perhaps these include individual HA units as they would not be able to induce hemagglutination and yet could be detected in antibody binding assays like SRID and ELISA. However, in immunogenicity assays they might be more like peptides as immunogens and require an effective adjuvant for inducing an antibody response. In an immunization study of the H7 vaccine in ferrets with and without an oil-in-water adjuvant vaccine performed after the poor immunogenicity in humans was known, immunogenicity was negligible with vaccine alone but was significantly improved when AS03 adjuvant was used (R. Webby, personal communication).

The classical stellate structures of HA and NA as described by Laver in the 1970s were prominent structures in the A/VN/04 (H5N1) vaccine and the A/CK/G9/99 (H9N2) vaccine. The A/VN/04 (H5N1) vaccine appeared intermediate in immunogenicity (Table 1); however, the A/Indo/05 vaccine was of similar immunogenicity without a clear morphologic association. The degree of prevalence of morphologic units with no distinct structure (Table 4) did not appear to relate to immunogenicity.

The studies reported here have suggested that, despite the variable and sometimes poor immunogenicity in humans, the inactivated avian influenza A virus vaccines all contain conformationally intact HA proteins capable of inducing some HAI antibody. Uptake, processing and presentation to human T cells and the state of the HA proteins, as determined in antibody binding assays and gel analyses, appear normal. These findings are reassuring for the potential of making useful avian influenza A vaccines. All the vaccines evaluated by us in this study are subunit vaccines that resulted from detergent treatment and other proprietary manipulations. It is known that these processes do not always split virus completely. An example of this was seen with one of the monovalent p2009 (H1N1) vaccines evaluated in this study which contained residual intact virus particles. The poorest immunogenicity was exhibited by the H7 vaccine which was predominantly small units that may be HA and NA units. One of the intermediate immunogenicity vaccines (H5N1) contained typical stellates. These findings suggest that the morphology of the vaccines may have influenced immunogenicity of these subunit vaccines in humans.

Vaccine morphology has been known to relate to immune responses to influenza vaccines in animal models and humans for decades. In general, whole virus vaccines have been more immunogenic than subunit vaccines and the smaller the subunit, the less immunogenic is the vaccine in naïve, unprimed hosts, best demonstrated in humans with a single dose [33], [50]. At the peptide/epitope level, an adjuvant is generally required to elicit good responses. Use of subunit vaccines became common after the extensive immunogenicity studies of A/New Jersey/76 (H1N1) vaccines in humans in 1976 [33], [50]. This trend was primarily for the reduced reactogenicity of subunit vaccines as whole virus vaccines were frequently shown to be more immunogenic than subunit vaccines but also more reactogenic. Proponents for superiority of whole virus vaccines continue to report findings in animal models and whole virus influenza vaccines are the products distributed by many companies throughout the world.

An effect of morphology on immunogenicity of influenza vaccines in humans was clearly demonstrated in the publications of Laver and Webster in the 1970s. Laver successfully removed the HA and NA from virus particles and purified the subunits [51]. The resulting subunits formed stellates when the hydrophobic ends attached to each other in aqueous solution. Those subunit vaccines, called hanaflu, were reduced in immunogenicity compared to whole virus vaccines in hamsters unless some whole virus was included. Webster and Laver showed that the whole virus could be influenza B even though the hanaflu vaccine evaluated was influenza A [52]. This finding could relate to immunogenicity of seasonal subunit trivalent vaccines which commonly contain virus particles as the type and subtype of the particles is unknown for these immunogenic vaccines. When the hanaflu stellate vaccines were tested with and without influenza B whole virus in humans, no enhancement in antibody responses was seen although the subjects were primed adults [52]. However, when the hanaflu/whole virus concept was tested in subjects unprimed for A/NJ/76 (H1N1) and A/USSR/77 (H1N1), enhancement was demonstrated when the hanaflu stellate vaccine included some whole virus [53], [54]. The intermediate immunogenicity of an H5N1 vaccine that contained stellate structures is compatible with the reduced immunogenicity seen in unprimed subjects with the pure hanaflu “stellate” vaccines by Webster and Laver.

Exceptions to a uniform proposal for whole virus vaccine superiority for immunogenicity in humans are in the varied immunogenicity reports in the clinical trials in 1976 and 1977 and for recent trials with avian influenza A inactivated whole virus vaccines [16]–[24], [27], [30], [33], [50]. The reported experience with avian monovalent whole virus vaccines without adjuvant is insufficient for conclusions on immunogenicity of whole virus versus split product vaccines without adjuvant. Two different A (H5N1) studies apparently using the same whole virus vaccine with and without alum adjuvant varied in reported immunogenicity [19], [20]. The only other identified trial of A (H5N1) whole virus vaccine without adjuvant was with A (H5N1) in healthy adults and 69% achieved HAI titers of ≥1∶40 from a dosage of 7.5 µg of HA [24]. A trial with an A (H9N2) whole virus vaccine is notable for finding low immunogenicity [27]. Most reported avian influenza whole virus vaccine trials were with alum adjuvant; the reported range of percent achieving HAI titers of ≥1∶40 is 20–64% for 5–7.5 µg and 45–86% for 15 µg of HA [16]–[23]. Reported percentages achieving ≥1∶40 HAI for 7.5 and 15 µg HA of subunit vaccine with alum adjuvant are 1–43% for 5–7.5 µg HA and 2–44% for 15 µg HA [7]–[9]. These findings suggest but do not prove a generally greater immunogenicity for whole virus vaccines; however, they are inferior to those reported for subunit vaccine with oil-in-water adjuvant [3], [8], [11], [14], [15], [28].

Limitations of the current study include the limited number of vaccines and different manufacturers evaluated, the incomplete evaluations of the conformational state of the HAs because of lacking some required reagents, the fact that the SRID concentrations were provided by different manufacturers who did not always prepare and use reagents as originally described by Schild, et al., and lack of a single starting antigen vaccine constructed to exhibit different morphologies and HA titers for correlating with immunogenicity [1]. Additionally, although the immunogenicity reports in the literature generally support the importance of vaccine morphology in inactivated avian influenza A vaccines for immunogenicity, there are inconsistencies. The clinical trial data compared were developed in a number of different laboratories and HAI titers are known to exhibit considerable variation between laboratories [55], [56].
